# The pedicled thoraco-umbilical flap: A versatile technique for upper limb coverage

**DOI:** 10.4103/0970-0358.59274

**Published:** 2009

**Authors:** Sharad Mishra, Ramesh Kumar Sharma

**Affiliations:** Department of Plastic Surgery, Postgraduate Institute of Medical Education and Research, Chandigarh, India

**Keywords:** Upper limb soft tissue defects, thoraco-umbilical flap, periumbilical perforators, deep inferior, epigastric artery

## Abstract

Injuries to upper limb has been on the increase and is invariably associated with significant soft tissue loss requiring a flap cover. Local tissue may not be available for cover in a majority of situations, necessitating import of tissue from a distant source. We have utilized the thoraco-umbilical flap taken from the trunk for this purpose. This flap is based on the perforators of the deep inferior epigastric artery that are maximally centred on the periumbilical region. This flap was used in 83 patients. The patients were observed for at least 3 weeks and any flap or donor site complications were recorded. The patients were again followed up at 3 months interval and the donor site scar was assessed. The flaps survived in 81 patients; there was marginal flap necrosis in five patients and partial flap necrosis in two patients. None of these patients required any additional procedure for coverage. The flap is technically easy to plan, almost effortless to drape around upper limb defects, with no significant donor site morbidity and also the post operative immobilization was fairly comfortable. The thoraco-umbilical flap thus is a very useful technique for coverage of the upper limb and is recommended as a first line flap for this purpose.

## INTRODUCTION

The injured upper limb on many occasions necessitates cover with a full thickness flap from a distant region. Even in the modern era of micro-vascular surgery, pedicled distant flaps still have a place in the soft tissue reconstruction of the hand and forearm. In the past, the abdominal skin was used based on sub-dermal random blood supply, and the flap size was restricted by the length to breadth ratio. The description of the axial groin flap revolutionized the management of hand injuries[1] and has remained a workhorse for management of hand injuries. However, this flap has some limitations; mainly the dependent position of the limb that leads to flap oedema. The groin flap may be insufficient if large areas of hand and adjacent forearm need to be covered. The coverage of elbow defects with this flap can be quite cumbersome for the patient. So we were looking for a flap that can cover the hand and adjacent forearm defects, has consistent anatomy, is easy to harvest and is positioned higher up in the trunk so that the postoperative oedema is less

Taylor and Boyd[2] in 1975 described the vascular territory of the deep inferior epigastric artery and suggested the use of the flap based upon these vessels. As the predominant vascular connections were oriented upward and laterally, they suggested that a thoraco-umbilical axis of the flap is the most suitable.[3] The flap has been used as both pedicled and free flap under varied clinical situations. It has been used for upper limb and groin reconstruction as a pedicled flap.[4–13] Although a hardy and versatile flap, it has been relegated to the category of ‘second choice flaps’ as there have been only a few reports in the literature regarding its usefulness in clinical setting.[10,13] This paper describes our experience with the thoraco-umbilical flap.

## MATERIAL AND METHODS

This flap has been used in 83 patients. The demographic details are given in Table 1. The defect size varied from 20 cm^2^ to 288 cm^2^ [Table 1]. The procedure was assessed for technical ease of harvesting during surgery. The operating surgeon was given a four-point questionnaire for every procedure and responses were noted on a Likert scale.[14]

**Table 1:** Demographic details and defect size

Age; no. of patients	<40;	>40;	
	61	22	
Sex (n)	Male	Female	
	(70)	(13)	
Size of defect	<100 cm^2^	101 – 200	>200 cm^2^
	33	cm2 39	11

All patients remained under clinical observation for a minimum period of 3 weeks. Immediate and early complications including flap loss, marginal necrosis, infection, dehiscence, etc were noted. Patients were also assessed at 3-month follow up visit when delayed complications such as complications of immobilization e.g. stiffness, pressure ulcers etc. were noted. Donor site complications were also recorded including donor site infection, dehiscence, unsightly scar and donor site hernia. At 3-month follow up visit, donor site scar was assessed using patient and observer scar assessment scale.

### Vascular Anatomy of the Flapth

The vascular anatomy of the paraumbilical region is well known.[3–10] The skin island of the thoraco-umbilical flap is supplied by paraumbilical perforators from the deep inferior epigastic vessels. The largest perforator is located at approximately 2 cm from the umbilicus, and directs toward the inferior angle of the scapula, anastomoses with the posterior intercostal artery and angulates 45° with the midline.[10] In a recent microdissection study, El-Mrakby *et al*.[11] investigated the course of paraumbilical perforator vessels. They followed the course of the musculocutaneous perforator vessels in the deep subcutaneous fat and then superficial to the Scarpa fascia until their final destination. It was demonstrated that superficial to the Scarpa fascia, the direct perforators turn to take a course parallel to the skin, where they started to divide for forming subdermal plexus. These perforators were also found to be anastomosing with the superficial vessels in the abdominal wall.

### Operative Technique

**Figure 1:**
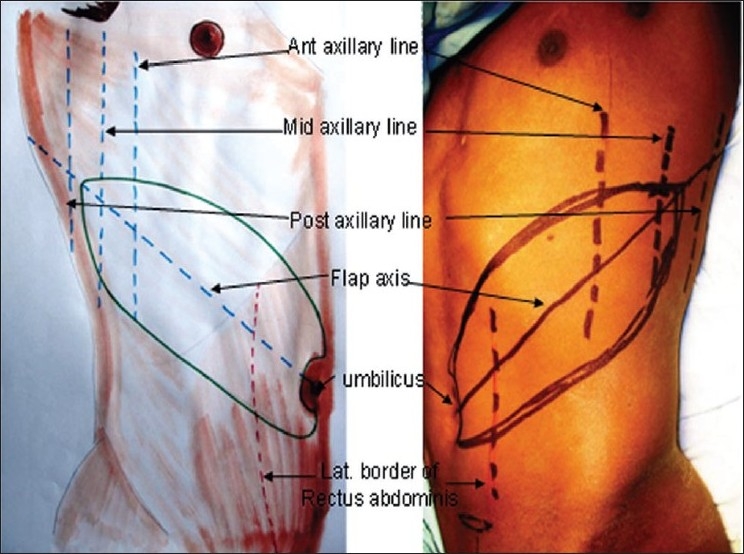
Marking of the flap

**Figure 2:**
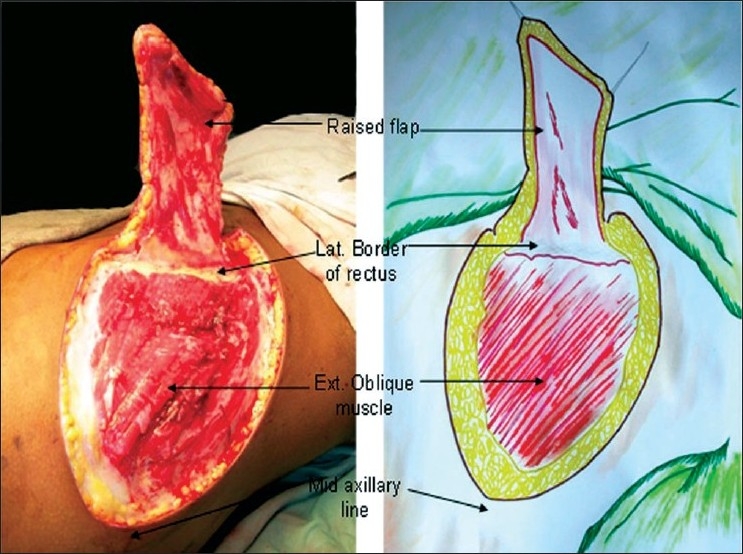
Medial limit of the flap; the perimysium of the underlying muscles is raised with the flap

**Figure 3-4:**
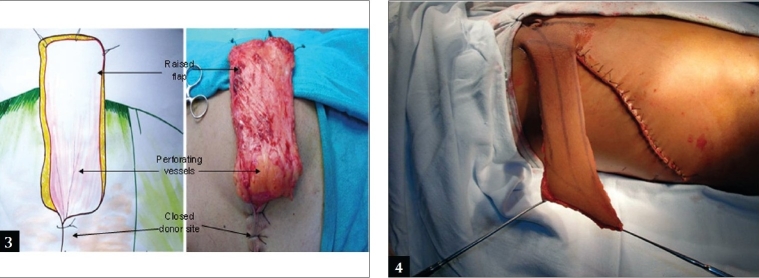
The donor site has been closed primarily

All procedures are done under general anaesthesia. A sand bag is placed under the lower ribs on the same side. The axis of the flap is marked as a line extending superiorly and laterally from the umbilicus to the tip of the scapula [Figure 1]. The mid and posterior axillary lines are marked. Pinching the skin assesses the elasticity of the skin and hence the width of the flap. The flap is raised from distal (lateral) to proximal (medial). It is elevated just superficial to the underlying musculature, to include not only the subdermal plexus but also the less important vessels within the fat and the flimsy fascia overlying the abdominal muscles. The dissection ceases about 2-3 cm medial to the lateral border of the rectus muscle or at any point of time if the flap is found to be adequate in length [Figure 2]. However, if more length of the flap is required, then the dissection continues medially, the rectus muscle is divided and the flap is thus based on the inferior epigastric artery only. The surrounding tissues are undermined and the donor site is closed primarily [Figure 3, 4]. The arm is immobilized on the patient's side with the help of adhesives and a pillow is placed beneath the elbow. The flap division and final inset are performed 3 weeks later.

**Figure 5:**
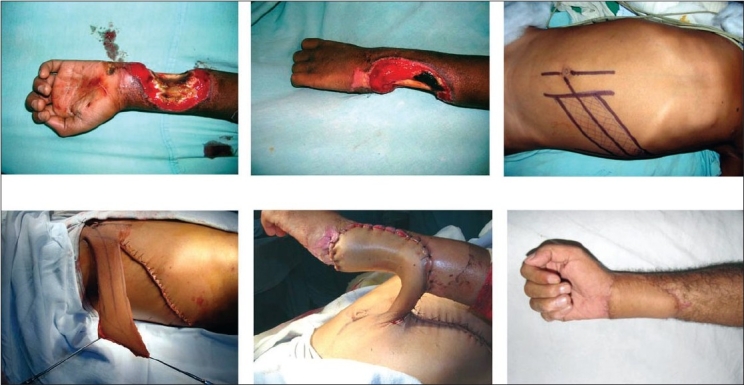
Electrical burns -defect in the forearm and wrist, execution of the flap

**Figure 6:**
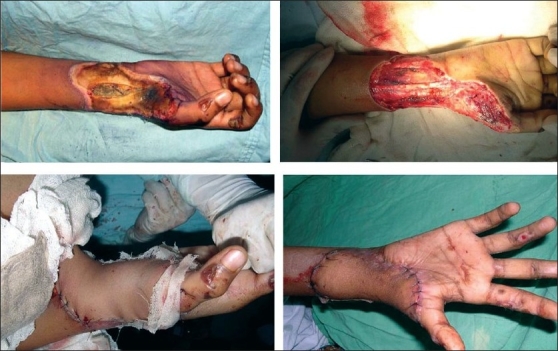
Another example of use of the flap

The planning and execution of the flaps are shown in two patients in Figures 5 and 6.

## RESULTS

**Figure 7:**
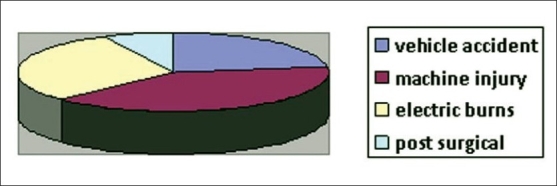
Aetiology of the defects

**Figure 8:**
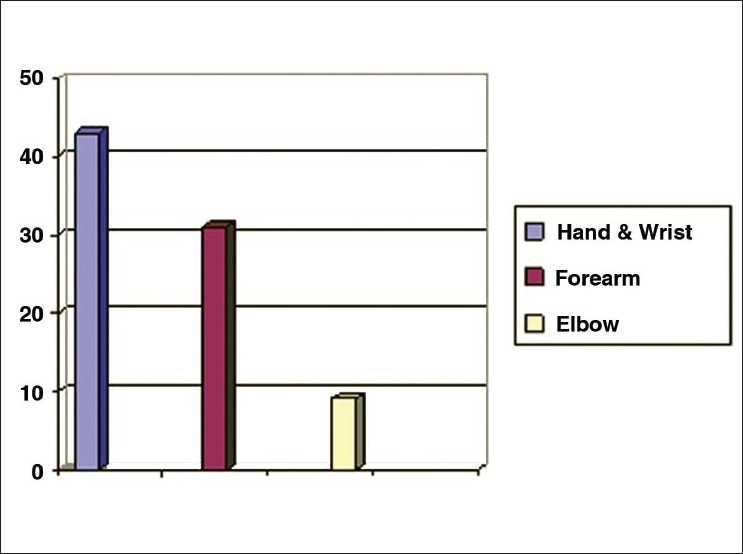
Location of the defects

This flap was done in 83 patients. Seventy patients were males and 13 were females. The age group was 4-56 years. Aetiology of injury included vehicular accidents in 28 patients, machine injury in 24 patients, electrical burns in 25 patients and post-surgical in 6 patients [Figure 7]. The flap was elevated till the mid-axillary line in 72 patients. In 11 patients, it was raised till the posterior axillary line [Table 2]. The location of the defect included hands, forearm and the elbow region [Figure 8].

**Table 2:** Flap size and lateral limit

*Flap size*	*Number*	*Lateral limit*
> 100 cm^2^	0	_
101–200cm^2^	44	Mid-axillary line
201–300cm^2^	32	Mid-axillary line
301–400cm^2^	7	Posterior axillary line

During initial post-operative, assessment wound cover was found to be satisfactory in all cases. Two flaps showed signs of venous congestion. One of these flaps was in a patient who developed cardiac arrest and prolonged hypotension. The other one was in a patient of old electric burns. At 3-week assessment only these two flaps failed.

Partial flap loss was found in two cases and marginal necrosis in five cases. Complete flap detachment occurred in two cases that required another salvage procedure. Partial flap detachment occurred in seven cases, due to marginal necrosis (five cases) and severe infection (two cases) that required resuturing [Table 3]. One patient was a psychiatric patient; he pulled off his flap on seventh postoperative day. The flap was then given a re-inset. Recipient site infection occurred in 11 cases out of whom 9 could be controlled with antibiotics and local wound care. Two flaps had partial dehiscence that required secondary suturing. Joint stiffness occurred in all cases but resolved with post-operative physiotherapy.

**Table 3:** Characteristics of flaps with marginal necrosis

*Number*	*Cause*	*Delay in surgery (days)*	*Bed status*	*Flap size (cm)*	*Lateral limit*
1	RSA	3	Contaminated	23 × 11	MAL
2	EB	10	Contaminated	20 × 12	MAL
3	RSA	1	Contaminated	32 × 10	PAL
4	EB	16	Infected	25 × 15	MAL
5	RSA	0	Contaminated	32 × 12	PAL
6	EB	15	Contaminated	30 × 12	PAL
7	EB	20	Infected	28 × 10	PAL
8	EB	12	Contaminated	27 × 12	MAL
9	RSA	2	Contaminated	30 × 14	PAL

RSA: Road-side accident, EB: Electrical burns, MAL: Mid-axillary line, PAL: Posterior axillary line

Donor site infection occurred in 10 cases with partial suture line dehiscence. Eight of these healed by secondary intention, while two required split skin grafting. Abdominal wall hernia was not seen in any patient.

The flap was technically easy to execute at every step - identification of its anatomical landmarks, identification of the sub-fascial plane and draping the contours of the injured upper limb. The flap was not bulky in most of the patients. Fortunately, majority of the patients were male labourers and were quite skinny. Even in female patients the part of the flap that is finally transferred onto the defect is not very bulky as this part of skin is from the lateral part of trunk.

**Figure 9:**
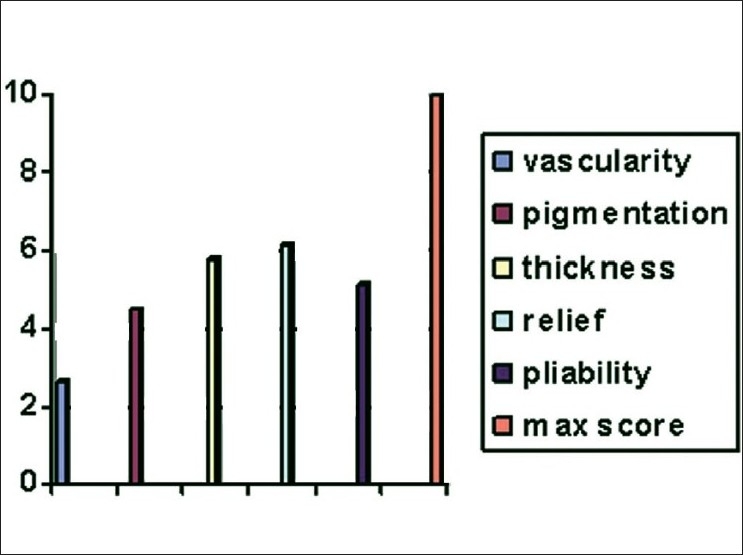
Observer assessment of scar chart

Scar assessment was done at a follow-up period of 3 months using the observer scar assessment scale (range 5: best scar possible – 50: worst scar possible; Figure 9) and patient scar assessment scale (Range 6: no complaints, normal skin – 60: serious complaints, scar very different from surrounding skin Table 4).

**Table 4:** Patient scar assessment

*Criteria*	*Score*
Is the color of scar different?	4.2
Is the scar more stiff?	4.0
Is the thickness of scar different?	4.6
Is the scar irregular?	5.5
Total score	18.3

On observer scale, the total score ranged from 21 to 35, with average being 24.4. On patient scar assessment, the average score was 20.7 (range 16-32).

## DISCUSSION

An axial flap is one containing a known vessel oriented along the axis of the flap. When the skin is supplied by vessels radiating from a single source rather than by an even distribution of direct musculocutaneous perforators, then a degree of ‘axiality’ is imparted to any flap containing such a radiating vessel in its long axis.[12] The basis for the thoraco-umbilical flap is the radiating nature of the dominant periumbilical perforators originating from the deep inferior epigastric artery. It would seem logical that if a flap was designed with its base in the umbilical region and its axis orientated along the major interconnections with the intercostal system, it would behave as an axial flap, allowing an advantageous length-to-breadth ratio and would provide the means of covering soft tissue defects in the upper extremity.

The thoraco-umbilical flap was first described by Taylor and Boyd.[4] They deduced that a large flap could be designed in many directions along axes that radiate from the umbilicus. The best flap appeared to be one planned along the axis between the umbilicus and the inferior angle of the scapula, running parallel to the ribs. By including a disc of anterior rectus sheath together with underlying muscle, the para umbilical perforators could be preserved. Dissection of the vascular pedicle toward the groin, with or without the rectus muscle, permitted the skin flap to rotate through a wide arc. In 1984, Taylor *et al*.[5] described their clinical experience with 18 patients treated with various combinations of deep inferior epigastric (thoraco-umbilical) flap. There were 70 male patients and 13 female patients in our study. This is in contrast with the previous study by Yilmaz *et al.*[13] wherein majority of the patients were females (72.7%). We believe that the male preponderance in our study is because of the fact that males are more exposed to the upper limb trauma in our scenario. The operative technique is easily learned with well-defined anatomical landmarks and requires average skill for raising the flap. It is simple, speedy and safe. All the flaps had a large length to breadth ratio, and all were elevated at least to the mid-axillary line. Out of the 11 patients whose flaps were elevated till posterior axillary line, 4 had partial necrosis involving the distal portions.

This flap is in fact a ‘perforator plus’ flap[15] and the base of the flap contains perforators from the deep inferior epigastric artery that are centered around the periumbilical region. The distal part of the flap is from the thin and pliable thoracic skin and appears extremely suitable for coverage of upper-limb defects. The proximal part of the flap is rather bulky but tends to settle with time after final inset in most of the patients. We incorporate the subcutaneous fat and fascia to ensure that the vessels are included. Post-operatively, upper limb with the attached flap could be maintained at a reasonably elevated level and did not require strict positioning. The arm rests in a comfortable natural position. It also helps in preventing oedema and facilitates physiotherapy. Patients can also wear their clothes comfortably as compared to the groin flap. In our study, 95% of the flaps were successful and were able to cover the intended areas. Since majority of our flaps were done in emergency setup and were used for coverage of contaminated wounds, infection was a problem. Also, because the pedicle of the flap could neither be tubed nor grafted, discharge from this site was common. However, such infections were usually self-limiting, and only two patients had partial flap dehiscence due to infection. Out of 10 flaps that were elevated beyond mid-axillary line, 4 developed marginal necrosis and partial flap dehiscence. These were re-advanced and were able to provide stable coverage. The first harvest acted as a delay and we were able to recruit the adjoining angiosomes by delay. There were two cases of complete flap failure. Both of these patients had partial flap necrosis resulting in complete flap detachment. One patient had prolonged post-operative hypotension requiring cardio pulmonary resuscitation and ventilatory support. Second patient was a case of old electric burns.

The donor site closure was not difficult and we were able to close the donor site primarily in all cases. There was always some tension at the suture line at the time of closure but it usually healed well. Two patients of electric burns for whom wide flaps were harvested, required split skin grafting after suture line dehiscence.

An unsightly scar has been suggested as the main drawback of this flap. In our study, however, none of the patients had any complaints about the scar except one patient who requested scar revision for cosmetic purpose. Remaining patients were satisfied with the scar. On objective parameters, however, observers assessing the scar quality were more critical of it than the patients themselves. The observer assessment is similar to the ones reported earlier.[6,13] Patients were generally unwilling to undergo further surgical procedure for scar revision, as it could be well concealed in clothing.

The flap fares quite well as compared to the commonly used groin flap. The groin flap based on the superficial circumflex iliac artery is a very useful flap for hand defects, but it is difficult to use this flap for extensive forearm defects because of the inferior and uncomfortable position of the flap. Moreover, the dependant position of the hand harnessed in a groin flap makes it oedematous, and the post operative physiotherapy more cumbersome. The narrow pedicled of the thoraco-umbilical flap enables a significant comfort for the patient in terms of upper-extremity movements. During the waiting period of 3 weeks for flap detachment, the hand and forearm are in a better physiologic condition than with the groin flap, and early mobilization of the hand is possible with the use of this flap.

## CONCLUSION

The thoraco-umbilical flap is a very useful flap for the coverage of upper-limb defects. A fairly large flap can be harvested and the donor site can be closed primarily in majority of the patients. There is no need to isolate the vascular pedicle, and the dissection is quick and systematic. The flap remains in an elevated position postoperatively and there is minimal oedema and congestion. Although the donor site scar is not concealed as in groin flap, majority of our patients accepted the donor scar because our traditional dresses conceal it anyway. This flap can become our workhorse for upper extremity defects, especially in an emergency setting, where defect size is large and/or emergency free flap is not feasible. It can be raised with ease and speed, and is a reliable flap.

## References

[1] McGregor IA, Jackson IT (1972). The groin flap. Br J Plast Surg.

[2] Taylor GI, Daniel RK (1975). The anatomy of several free flap donor sites. Plast Reconstr Surg.

[3] Boyd JB, Taylor GI, Corlett R (1984). The vascular territories of the superior epigastric and deep inferior epigastric system. Plast Reconstr Surg.

[4] Taylor GI, Corlett R, Boyd JB (1983). The extended deep inferior epigastric flap: A clinical technique. Plast Reconstr Surg.

[5] Taylor GI, Corlett RJ, Boyd JB (1984). The versatile deep inferior epigastric flap. Br J Plast Surg.

[6] Boyd JB, Mackinnon SE (1989). An evaluation of pedicled thoraco umbilical flap in upper extremity reconstruction. Ann Plast Surg.

[7] Fan QS (1987). The anatomy and application of the thoracoumbilical flap. Chin J Microsurg.

[8] Fan QS (1992). The thoraco-umbilical flap for a large skin defect on hand and forearm. Chin J Hand Surg.

[9] Fan QS (1987). The anatomy and application of the thoracoumbilical flap. Chin J Microsurg.

[10] Zhang XQ, Wang SD, Fan QY, Mao BA, Zhou Y, Zhang MH (2004). Thoracoumbilical flap: experience with 33 flaps. J Reconstr Microsurg.

[11] El-Mrakby HH, Milner RH (2002). The suprafascial course of the direct paraumbilical perforators vessels. Plast Reconstr Surg.

[12] Cormack GC, Lamberty BG (1986). Cadaver studies of correlation between vessel size and anatomical territory of cutaneous supply. Br J Plast Surg.

[13] Yilmaz S, Saydam M, Seven E, Ercocen AR (2005). Paraumbilical perforator-based pedicled abdominal flap for extensive soft-tissue deficiencies of the forearm and hand. Ann Plast Surg.

[14] Rensis L (1932). A Technique for the Measurement of Attitudes. Archives of Psychology.

[15] Sharma RK, Mehrotra S, Nanda V (2005). The perforator plus flap: a simple nomenclature for locoregional perforator based flaps. Plast Reconstr Surg.

